# Second Trimester Fetal Loss Due to *Citrobacter koseri* Infection: A Rare Cause of Preterm Premature Rupture of Membranes (PPROM)

**DOI:** 10.3390/diagnostics12010159

**Published:** 2022-01-10

**Authors:** Maria Paola Bonasoni, Giuseppina Comitini, Mariangela Pati, Giuseppe Russello, Loredana Vizzini, Marcellino Bardaro, Pietro Pini, Roberta Marrollo, Andrea Palicelli, Giulia Dalla Dea, Edoardo Carretto

**Affiliations:** 1 Pathology Unit, Azienda USL-IRCCS di Reggio Emilia, 42123 Reggio Emilia, Italy; Andrea.Palicelli@ausl.re.it; 2 Department of Obstetrics & Gynaecology, Azienda USL-IRCCS di Reggio Emilia, 42123 Reggio Emilia, Italy; Giuseppina.Comitini@ausl.re.it (G.C.); Mariangela.Pati@ausl.re.it (M.P.); 3 Clinical Microbiology Laboratory, Azienda USL-IRCCS di Reggio Emilia, 42123 Reggio Emilia, Italy; Giuseppe.Russello@ausl.re.it (G.R.); Loredana.Vizzini@ausl.re.it (L.V.); Marcellino.Bardaro@ausl.re.it (M.B.); Pietro.Pini@ausl.re.it (P.P.); Roberta.Marrollo@ausl.re.it (R.M.); Edoardo.Carretto@ausl.re.it (E.C.); 4 Pathology Unit, “Maggiore della Carità” Hospital, 28100 Novara, Italy; gdalladea12@gmail.com

**Keywords:** *Citrobacter koseri*, preterm premature rupture of membranes, chorioamnionitis, vertical transmission

## Abstract

*Citrobacter koseri* is a facultative anaerobic, motile, non-spore-forming Gram-negative bacillus, which belongs to the family of Enterobacteriaceae. Severe infections due to *Citrobacter* spp. have been reported in the urinary tract, respiratory airways, intra-abdominal organs, skin and soft tissue, eye, bone, bloodstream, and central nervous system. In newborns, *C. koseri* is a well-known cause of meningitis, cerebral abscesses, brain adhesions, encephalitis, and pneumocephalus. Infection can be acquired through vertical maternal transmission or horizontal hospital settings; however, in many cases, the source is unknown. Preterm premature rupture of membranes (PPROM), caused by *C. koseri*, has rarely been described. Herein, we describe a case of PPROM at 16 weeks and 3 days of gestation, leading to anhydramnios. The parents opted for legal termination of the pregnancy, as the prognosis was very poor. *C. koseri* was isolated postmortem from a placental subamniotic swab and parenchymal sample, as well as fetal blood and lung. To the best of our knowledge, this is the first case of early second-trimester PPROM in which *C. koseri* infection was demonstrated.

## 1. Introduction

*Citrobacter koseri* (formerly *Citrobacter diversus*) belongs to the family of Enterobacteriaceae, and is a facultative anaerobic, motile, non-spore-forming Gram-negative bacillus. *Citrobacter* species are commonly found in water, soil, sewage, food, and occasional colonizers of the gastrointestinal tract of animals and humans. *Citrobacter* spp. may cause severe infections in the urinary tract, respiratory airways, intra-abdominal organs, skin and soft tissue, eye, bone, bloodstream, and central nervous system [1]. 

In neonates, *C. koseri* infection is responsible for meningitis, cerebral abscesses, brain adhesions, encephalitis, ventriculitis, and pneumocephalus [2,3,4,5,6,7]. The bacterium is acquired from vertical maternal transmission at delivery or through horizontal nosocomial spread, but, in most cases, the origin remains unknown [3]. 

Vertical transmission is more likely if the symptoms occur in the first days of life, although isolation of the pathogen in the mother is uncommon. In general, *C. koseri* infection in newborns occurs sporadically, and it is classified in early onset (between 5 and 12 days of age) and late onset (around 4–5 weeks of age) [3]. 

Clinical manifestations of central nervous system infection are similar to other pathogens, such as poor feeding, vomiting, hypotonia, seizures, lethargy, fever, and bulging fontanelle. The main complication is meningitis, due to a 32 kDa protein present in the external membrane of the bacteria. Brain abscesses are extremely frequent in *C. koseri* meningitis, occurring in almost 80% of cases, compared to 1% of other etiologies. Conversely, the mortality rate in *C. koseri* meningitis is around 30%, and neurological sequelae are reported in more than 80% of cases [3,4,5]. Infant management must be prompt, and includes clinical evaluation, blood and urine cultures, and lumbar puncture for cerebrospinal fluid examination. Antibiotic therapy must be given according to antimicrobial susceptibility testing results. Serial magnetic resonance imaging (MRI) is the gold standard in detecting early cerebral complications [4]. 

Although *C. koseri* infection in infants is well recognized, it has rarely been described in prenatal settings as a cause of preterm premature rupture of membranes (PPROM) [8]. 

We report a new case of PPROM at 16 weeks and 3 days of gestation, which resulted in legal termination of the pregnancy, due to anhydramnios and adverse outcomes. *C. koseri* was isolated postmortem from the placental swab and tissue, as well as fetal blood and lung. To the best of our knowledge, this is the first case of early second-trimester PPROM caused by *C. koseri* infection. 

## 2. Case Description

A 39-year-old woman was admitted to the emergency department of our institution for abundant vaginal bleeding and PPROM at 16 weeks and 3 days of gestation. The patient was nulliparous with a history of cervical insufficiency and recurrent spontaneous abortion (RSA). She had six previous miscarriages, and two of them underwent fetal autopsies at our institution. Both of them occurred at 19 weeks of gestation (wga), and the prenatal ultrasound (US) showed no anomalies and regular fetal growth, which was confirmed by the autopsy. Unfortunately, microbiological cultures were not performed. The first miscarriage presented with vaginal bleeding, despite a cervical prophylactic cerclage. Histologically, there was acute chorioamnionitis, but neutrophils were not found in the fetal tissues. The second miscarriage presented with PPROM and anhydramnios. Microscopy showed acute chorioamnionitis and chorionic vasculitis. Abundant neutrophils were found in the fetal lung within the bronchi and alveoli, as well as into the gastric, and small and large intestine contents. 

Regarding the current miscarriage, the US prenatal findings were within the normal limits. The patient had a prophylactic cervical cerclage (Shirodkar type) positioned 17 days before the PPROM. At admission, the vaginal swab and urine culture were negative. She was started on antibiotics according to the PPROM guidelines [9], and was treated with ampicillin (2 g intravenous injection every 6 h) for 2 days, which was then replaced with amoxicillin (1 g capsule every 8 h for 3 days, and reduced to 1 g capsule every 12 h for 3 days). However, despite the medical therapy, at day four of hospitalization, the fetal conditions worsened with anhydramnios and the patient opted for legal termination of the pregnancy. Labour was induced and a stillborn female baby was delivered at 17 wga. The patient was discharged two days after the procedure in good health and she was given chlorhexidine in vaginal ovules for one week. 

Postmortem examination revealed a nonmacerated fetus weighing 145 g and measuring 19 cm in crown–heel length. The other measurements were as follows: crown–rump length: 13 cm; foot length: 2.2 cm; head, chest, and abdominal circumference: 13 cm, 10.5 cm, and 9.5 cm, respectively. Overall, the anthropometric measurements were consistent with 17 weeks gestation [10]. External examination showed a normal female fetus with mild facial and nuchal edema. Internal examination disclosed organ congestion and moderate pericardial, pleural, and abdominal serous effusions. No congenital anomalies were found. Microscopic analysis revealed the presence of only rare intra-alveolar and gastric neutrophils. No other significant histological findings were noted. 

The placenta was received complete, weighing 96 g and measuring 11 × 9 × 2 cm. Macroscopically, the placenta was pale, with an intra-parenchymal hematoma measuring 2 × 1 × 1 cm. The membranes were mildly opaque. Microscopically, there was acute chorioamnionitis (AC), corresponding to a stage 2/3 and grade 1/2 maternal inflammatory response [11]. Acute necrotizing deciduitis was also identified. A recent intraparenchymal thrombohematoma was confirmed, as identified grossly.

After the autopsy, the subamniotic swab and placental tissue of the patient, as well as the blood (collected through intracardiac sampling), lung, and liver of the fetus, were sent to the microbiology laboratory. Autoptical samples were cultured on Columbia blood agar (CBA), McConkey agar (MCA), mannitol salt agar (MSA), and Sabouraud (SAB) agar (all these media were incubated at 36 °C in ambient air), as well as chocolate agar (CA, incubated at 36 °C in 5% CO_2_) and Schaedler agar (SA, incubated at 36 °C in anaerobic atmosphere). After 24 h, the CBA plates of fetal blood, lung, subamniotic swab, and placental tissue yielded 2–4 mm in diameter, smooth, low, convex, and moist colonies, which were also present on MCA, CA, and SA. No growth was observed on MSA and SAB. The colonies were identified as *Citrobacter koseri* using the MALDI-ToF technology (Bruker Daltonics, Bremen, Germany), Biotyper OC software version 3.1. Fetal liver cultures remained negative. Antimicrobial susceptibility testing was performed using Phoenix 100™ (Becton Dickinson, Franklin Lakes, NJ, USA) and showed a wild-type antibiotype, displaying that the isolates were resistant to aminopenicillins only.

## 3. Discussion

Neonatal infection caused by *C. koseri* is a well-known entity, albeit uncommon. It typically causes sepsis, meningitis, encephalitis, and brain abscesses. Premature infants are more susceptible, and the overall prognosis is very poor, with high morbidity and mortality. *C. koseri* colonization of the lower gastrointestinal tract, respiratory tract, and oral cavity may be responsible for horizontal transmission from healthcare workers in hospitals or in the community from family members. Vertical transmission from mother to infant may occur at delivery or perinatally [3]. A definite acquired prenatal infection has been documented in a few cases, in which the bacterium has been isolated from the placenta, the mother, or the newborn in a short time after birth [12,13,14,15,16,17,18,19,20,21,22,23,24,25]. 

In general, the neonatal epidemiology of *C. koseri* is still poorly understood, with few reports about nursery outbreaks. Therefore, the transmission, reservoirs of infection, risk factors, and frequency of asymptomatic colonization need further investigation [25,26,27,28]. An infant with *C. koseri* infection may be a carrier, and may spread the bacterium in nursery. During outbreaks, many asymptomatic newborns may also be present, with an estimated ratio of 1:25 [26]. Hand carriage by nursery personnel seems to be the main mode of transmission. The source of infection may be the personnel’s hands and/or infants’ umbilicus, and spreading passes on from the nurses’ hands or the infants’ umbilicus to other infants’ umbilicus. The fecal–hand route and environmental origin seem less likely, but possible [27,28]. In cases of nursery outbreak, nasopharyngeal, rectal, and umbilical cord cultures must be taken from the infants, as well as hand and rectal samples from the personnel. Environmental sources should also be investigated with swabs from sinks, incubators, and medical equipment. Infection control procedures must be applied immediately, with frequent handwashing, introduction of routine surveillance cultures, and exclusion of culture-positive staff. The last precaution is considered the most effective, as *C. koseri* can persist chronically on hands or in the gastrointestinal tract, and then be easily reintroduced into the nursery [28].

Precautions in the nursery are mandatory, as preterm infants are at a higher risk of *C. koseri* infection, and neurologic symptoms can be vague, even in cases of severe cerebral damage [3,29]. When the diagnosis of *C. koseri* meningitis has been established, serial neuroimaging must be planned. Ultrasonography can be used for the initial assessment, but MRI is the hallmark for detecting cerebral damage [4]. Although brain abscesses are the main complication, favorable outcomes have been reported [29,30]. Surgical drainage, associated with intravenous antibiotic therapy with meropenem and fosfomycin, should be the treatment of choice in eradicating the infection [29].

As already mentioned, although neonatal *C. koseri* infection is well known, PPROM and subsequent fetal death due to this microorganism have been fully documented in only two cases [8]. 

In the first case, the mother had a history of two weeks of vaginal discharge. At admission, the fetal membranes were protruding through the cervical os and an emergency cervical suture was inserted. Vaginal and urine cultures were negative. After 5 days, she was febrile and US detected intrauterine fetal death (IUFD) at 25 wga. The patient fully recovered after septic shock, in which *C. koseri* was isolated from the blood. The growth of the same bacterium was obtained from the placenta, and fetal blood, lung, liver, and spleen. Histologically, there was severe AC, and neutrophils were observed within the alveoli. Interestingly, in the brain, abundant Gram-negative bacilli were found in the lumen of the penetrating small blood vessels. 

The second case was PPROM and IUFD at 18 wga in an asymptomatic mother. Although urine culture isolated *Escherichia coli*, the placental swab and fetal lung yielded *C. koseri*. AC and rare pulmonary neutrophils were also observed. 

In our case, PPROM occurred at 16 wga and 3 days. The mother presented with abundant vaginal bleeding, but she had never complained of fever. A prophylactic cervical cerclage (Shirodkar type) was inserted 17 days before hospital admission. Other than the report by Chan [8], in the other two, the mothers had cervical cerclage [15,18]. It is highly likely that in all these patients, the cerclage might have been the initial source of infection, and then the bacterium spread through the ascending route. 

In our case, *C. koseri* was isolated postmortem from the following different samples: the placental subamniotic swab and tissue, and fetal blood and lung. Antimicrobial susceptibility testing was performed on the fetal blood and demonstrated intrinsic resistance of the bacterium only, i.e., aminopenicillins. The patient was initially treated with ampicillin by intravenous injection of 2 g every 6 h for two days. The in vitro inefficacy of this therapy might explain the *C. koseri* isolation from the placenta and fetus. 

Histologically, AC and scarce neutrophils were detected in the lungs and into the gastric content. Contrary to Chan’s observation [8], no bacilli, inflammatory response, or parenchymal damage were found in the brain. 

*C. koseri* has a particular brain tropism, and it has been studied in vitro and in animal models. The in vitro studies demonstrated that *C. koseri* is capable of invading human U937 macrophages and endothelial cells of cerebral capillaries [31,32]. In neonatal rats, *fliP* favors bacterial macrophage intake, cytokine expression, and brain abscesses [33]. This microorganism has fewer resistance genes than other bacteria, which makes it more susceptible to antibiotics [34]. HPI genes are responsible for pathogenicity; vasculitis of small blood vessels favors hemorrhagic necrosis, tissue destruction, and cavitation. Abscesses from *C. koseri* typically lack a fibrous capsule. Moreover, the bacteria invade meninges and ependyma, with subsequent parenchymal disruption and hydrocephalus [35,36]. 

In neonates, the neuropathology of *C. koseri* has been detailed in only three cases [35,37]. Similarly to the animal model, cerebral necrotizing vasculitis was the main feature, with *C. koseri* parietal infiltration of the small vessels [29]. Moreover, a 32 kD bacterial membrane protein seems to be involved in meningeal tropism, as it was isolated in cerebrospinal fluid [3]. 

In our case, cerebral involvement was not observed, either with bacteria or brain damage. This could be explained by the short time of infection and/or the fetal immature immune response. 

In conclusion, *C. koseri*, although rare, can cause PPROM in the early second-trimester and IUFD, through ascending infection and/or a hematogenous route. Particular attention should be given to patients with a cervical cerclage, as it may be the origin of infection. Therefore, vertical transmission of *C. koseri* as a potential source of infection should not be underestimated, especially in newborns. 

## Data Availability

The data presented in this study are available on request from the corresponding author.
